# The Role of Omega-3 Polyunsaturated Fatty Acids in Heart Failure: A Meta-Analysis of Randomised Controlled Trials

**DOI:** 10.3390/nu9010018

**Published:** 2016-12-30

**Authors:** Chunbin Wang, Bo Xiong, Jing Huang

**Affiliations:** Department of Cardiology, The Second Affiliated Hospital of Chongqing Medical University, Chongqing 400010, China; w2321@eyou.com (C.W.); xbndan@163.com (B.X.)

**Keywords:** brain natriuretic peptide, heart failure, meta-analysis, omega-3 polyunsaturated fatty acids

## Abstract

Many new clinical trials about the effect of omega-3 polyunsaturated fatty acids (PUFAs) in heart failure (HF) patients have shown inconsistent results. Therefore, a meta-analysis of randomised controlled trials (RCTs) was performed to determine the benefits of omega-3 PUFAs in HF patients. Articles were obtained from PubMed, EMBASE, and the Cochrane Library. RCTs comparing omega-3 PUFAs with placebo for HF were included. Two reviewers independently extracted the data from the selected publications. The *I*^2^ statistic was used to assess heterogeneity. The pooled mean difference and associated 95% confidence intervals were calculated, and a fixed or random-effects model was used for the meta-analysis. A total of nine RCTs involving 800 patients were eligible for inclusion. Compared with patients taking placebo, HF patients who received omega-3 PUFAs experienced decreased brain natriuretic peptide levels and serum norepinephrine levels. Although the left ventricular ejection fraction (LVEF) and clinical outcomes (Tei index, peak oxygen consumption) did not improve, subgroup analysis showed that the LVEF increased in dilated cardiomyopathy (DCM) patients. Overall, omega-3 PUFA supplements might be beneficial in HF patients, especially in DCM patients, but further studies are needed to confirm these benefits.

## 1. Introduction

Heart failure (HF) is a common and growing public health problem worldwide. At present, approximately 26 million people are living with HF in the world. In the United States, there were 5.8 million patients with HF in 2012, and this is expected to increase to 8.5 million by 2030 [1]. Despite advances in pharmacological and non-pharmacological therapies, the treatment of HF remains a challenge [2,3]. Omega-3 polyunsaturated fatty acids (PUFAs), including eicosapentaenoic acid (EPA), docosahexaenoic acid (DHA), and plant-derived alpha-linolenic acid (ALA), are mainly derived from marine sources such as fish oil, as well as from nuts and vegetable oils. Omega-3 PUFAs are widely consumed as nutritional supplements. Health authorities have long recommended these supplements to provide cardiovascular protective effects in patients with coronary heart disease (CHD) [4]. Substantial evidence from experimental and epidemiological studies has suggested that the intake of omega-3 PUFAs could be beneficial for HF patients [5,6,7,8]. The GISSI-HF trial showed that HF patients receiving long-term fish oil treatment showed reduced endpoints of mortality or hospitalizations for cardiovascular reasons [9]. However, many questions still remain. Further clinical studies are warranted to clarify the optimal dose, the differential benefit of EPA and DHA, and the mechanism of action. A previous meta-analysis performed in 2012 [10] indicated that omega-3 PUFAs could improve cardiac function and functional capacity in HF patients. However, since the publication of that analysis, there have been many new clinical trials about the effect of omega-3 PUFAs in HF patients with different outcomes and inconsistent results [11,12,13,14]. For this reason, to assess a greater number of studies and more comprehensively determine the benefits of omega-3 PUFAs in HF patients, we performed a meta-analysis of randomised controlled trials (RCTs) to systematically evaluate the effects of omega-3 PUFAs in HF patients. In our meta-analysis, we did not include the study by Ghio et al. [15] because the patients received rosuvastatin, and three recently new published studies were included in the present analysis. In addition, we investigated some new outcomes, such as brain natriuretic peptide (BNP), and serum norepinephrine (SNE) levels, Tei index, and peak oxygen consumption (peak VO_2_) for analysis.

## 2. Materials and Methods

This meta-analysis followed the recommendations of the Preferred Reporting Items for Systematic Reviews and Meta-Analysis (PRISMA) statement [16]. The International Prospective Register for Systematic Reviews (PROSPERO) number is CRD42016034197.

### 2.1. Literature Search Strategy

We attempted to identify all relevant published randomised studies investigating the effects of omega-3 PUFAs in HF patients. Two reviewers systematically searched for pertinent studies in PubMed, EMBASE, and the Cochrane Library until 1 August 2016. This search was restricted to English-language publications. The following search strategy was used: (heart failure) AND (Omega 3 OR PUFA OR fish oil OR marine oil OR cod liver oil OR *n*-3 fatty acid OR polyunsaturated fatty acid OR eicosapentaenoic acid OR docosahexaenoic acid OR EPA OR DHA) (Appendix A).

### 2.2. Study Selection

The titles and abstracts of the retrieved articles were independently scanned by two reviewers. The eligibility of these articles was further assessed by full-text evaluation by the same two reviewers. Disagreements between the reviewers were resolved by discussion. Studies were eligible for inclusion if they met the following criteria: (1) clinical trial with RCT design; (2) patients with HF; (3) supplementation with omega-3 PUFAs as a component of treatment; and (4) reporting any outcomes of BNP levels, left ventricular ejection fraction (LVEF), Tei index, peak VO_2_, or SNE levels.

### 2.3. Data Extraction

Two reviewers independently extracted the relevant data from the included studies, and the third reviewer was responsible for repeated research, with divergences resolved by discussion. The extracted information included: characteristics of included studies (title, first author, publication year, journal, country, corresponding address, study design, inclusion and exclusion criteria, and pertinent outcomes such as BNP, LVEF, Tei index, peak VO_2_, and SNE levels). If several articles were based on data from the same study, only the study that included the most complete data was included in our meta-analysis.

### 2.4. Assessment of Risk of Bias

The risk of bias for included RCTs was independently evaluated by two reviewers in accordance with the Cochrane risk of bias tool [16]. Disagreements were resolved by discussion. The quality evaluation was judged on the use of random sequence generation, allocation concealment, blinding of participants and personnel, blinding of outcome assessment, incomplete outcome data, selective reporting, and other potential sources of bias.

### 2.5. Statistical Analysis

All of the statistical analyses were performed using Stata 12.0 (Stata Corp., College Station, TX, USA) and RevMan software (version 5.3, Cochrane Collaboration, Oxford, UK). Pooled effects were presented as weighted mean difference (WMD) with confidence intervals (CIs) for measurements of the same parameter or standardised mean difference (SMD) for various measurements in the included studies. Heterogeneity was evaluated using the *I*^2^ test (*I*^2^ > 50% indicating significant heterogeneity). An inverse variance fixed-effect model was used to calculate the WMD or SMD with 95% CI if there was no significant heterogeneity among the included studies; otherwise, a random-effect model was chosen. Sensitivity analysis was used to determine the stability of the statistical results by excluding each study sequentially. In addition, publication bias was evaluated with the use of funnel plots. Statistical significance was set at *p* < 0.05.

## 3. Results

### 3.1. Eligible Studies

**Figure 1:**
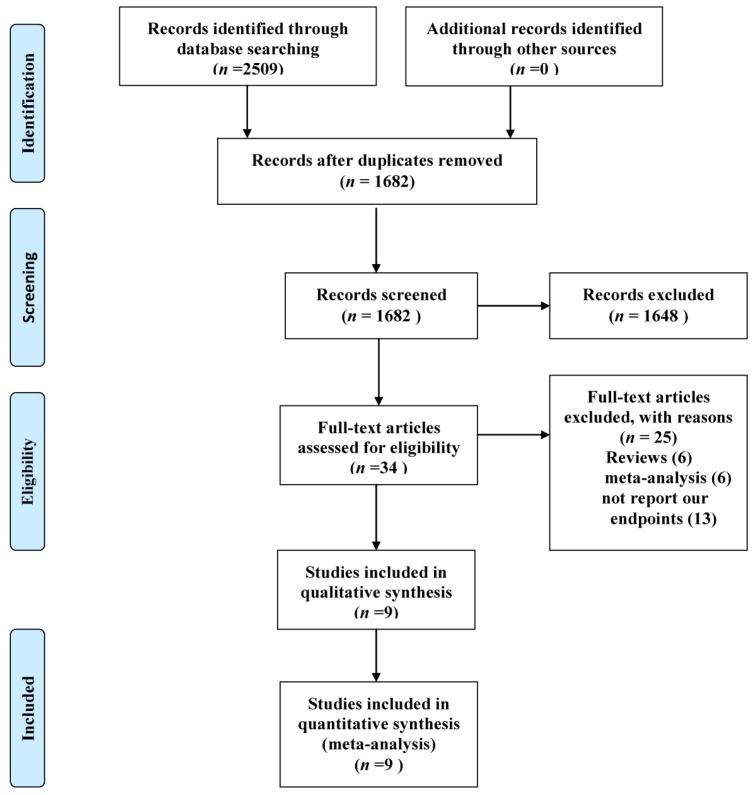
Preferred Reporting Items for Systematic Reviews and Meta-Analysis (PRISMA) flow diagram of study selection.

A total of 2509 (PubMed (1362), EMBASE (1103), and the Cochrane Library (44)) potentially relevant articles were found by searching the databases. The titles and the abstracts of the retrieved articles were examined, and 2475 were excluded. A full-text evaluation was performed for the remaining 34 articles, and 25 articles were excluded for the following reasons: reviews (6), meta-analysis (6), and did not report our endpoints (13). Therefore, nine studies were eligible for our meta-analysis. The process of the literature search and the reasons for exclusion are provided in Figure 1.

### 3.2. Study Characteristics

**Figure 2:**
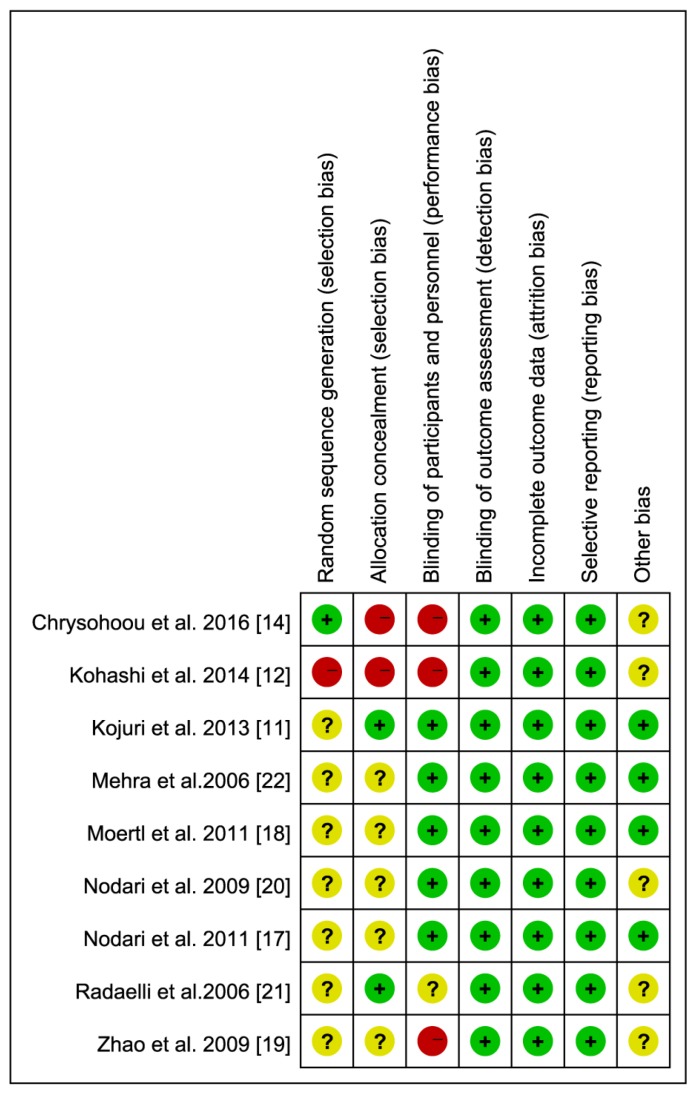
Risk of bias summary: Review authors’ judgements about each risk of bias item for each included study.

The remaining studies were nine clinical trials [11,12,14,17,18,19,20,21,22] and included a total of 800 patients, as described in Table 1 and Table 2. All nine studies were RCT designs published from 2006 to 2016. These studies reported that the participants were clinically and stable HF patients who received optimal medical therapy based on modern HF treatment strategies. The medication treatment was maintained during the follow-up interval. The aetiology of HF was dilated cardiomyopathy (DCM) in three studies [17,18,20], ischaemic cardiomyopathy (ICM) in two studies [11,21], and both ICM and DCM in four studies [12,14,19,22]. ICM was defined as a history of myocardial infarction with significant coronary artery disease (>70% luminal stenosis in at least two major coronary arteries) [12]. The mean baseline LVEF of the participants varied from 24% to 45%, and the New York Heart Association class was from I to IV. The dose of omega-3 PUFAs (calculated as the total dose of ALA, EPA, and DHA) ranged from 1 g/day to 4 g/day, with the ratio of EPA to DHA varying from 0.60 g/day to 1.53 g/day. The duration of the investigation varied from 3 to 12 months. None of these studies reported adverse events related to omega-3 PUFA supplementation. The quality score of the seven studies ranged from 3 to 5 (Figure 2). All of the studies were randomised and placebo controlled; only one study was performed in a single blind fashion [12].

### 3.3. Effect of Omega-3 PUFAs on BNP Levels

**Figure 3:**
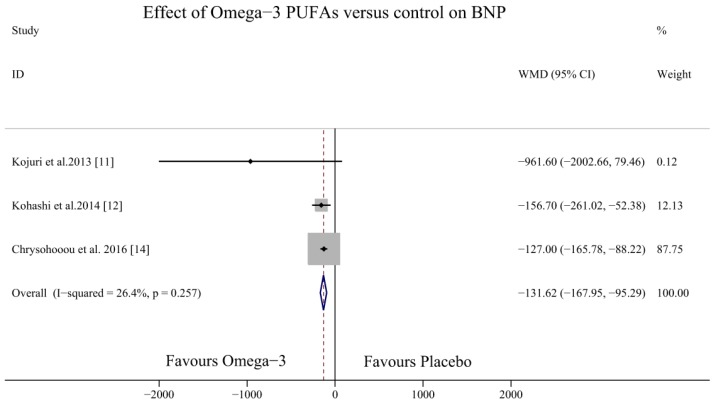
Forest plot depicting the effects of omega-3 PUFAs on BNP levels (WMD, weighted mean difference). Abbreviations: BNP, brain natriuretic peptide; PUFAs, polyunsaturated fatty acids.

**Figure 4:**
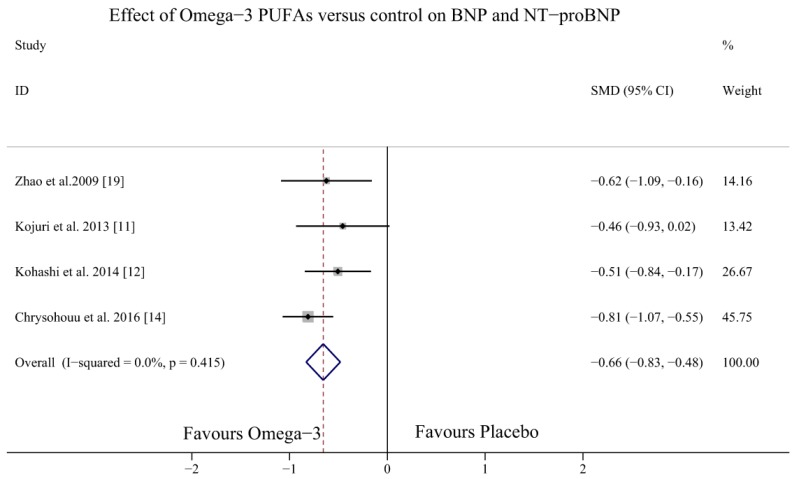
Forest plot depicting the effects of omega-3 PUFAs on BNP and NT-proBNP levels (SMD, standardised mean difference). Abbreviations: BNP, brain natriuretic peptide; NT-proBNP, N-terminal pro B-type natriuretic peptide PUFAs, polyunsaturated fatty acids.

Four studies investigated the effects of omega-3 PUFAs on the BNP levels in HF patients [11,12,14,19] (one study measured NT-proBNP levels [19] and three studies measured BNP levels [11,12,14]). The result demonstrated that omega-3 PUFAs could decrease BNP levels (WMD (weighted mean difference) = −131.62, 95% CI = −167.95 to −95.29; *p* = 0.257, *I*^2^ = 26.4%) (Figure 3) and NT-proBNP and BNP levels overall (SMD = −0.66, 95% CI = −0.83 to −0.48; *p* = 0.415, *I*^2^ = 0%) (Figure 4).

### 3.4. Effect of Omega-3 PUFAs on LVEF

**Figure 5:**
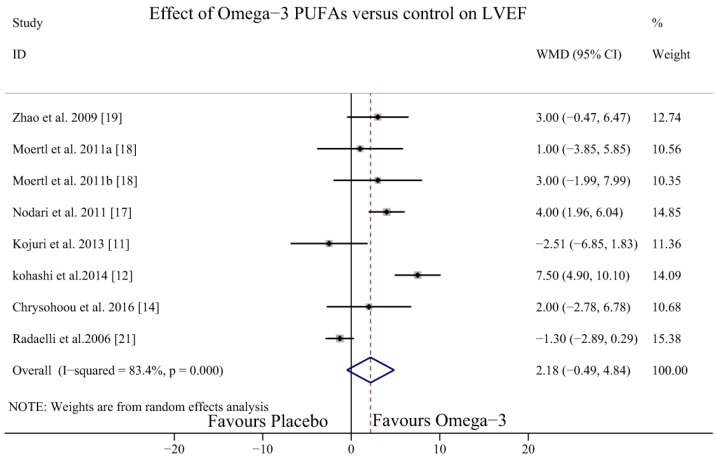
Forest plot depicting the effect of omega-3 PUFAs on LVEF (WMD, weighted mean difference). Abbreviations: LVEF, left ventricular ejection fraction; PUFAs, polyunsaturated fatty acids.

There were seven studies [11,12,14,17,18,19,21] with eight study arms testing the effect of omega-3 PUFAs on the LVEF of HF patients. Significant heterogeneity was detected among the study arms (*I*^2^ = 84.3%, *p* = 0.00); therefore, the random effect model was applied. The results showed that omega-3 PUFAs could not increase the LVEF (WMD = 2.18, 95% CI = −0.49 to 4.84) (Figure 5).

**Figure 6:**
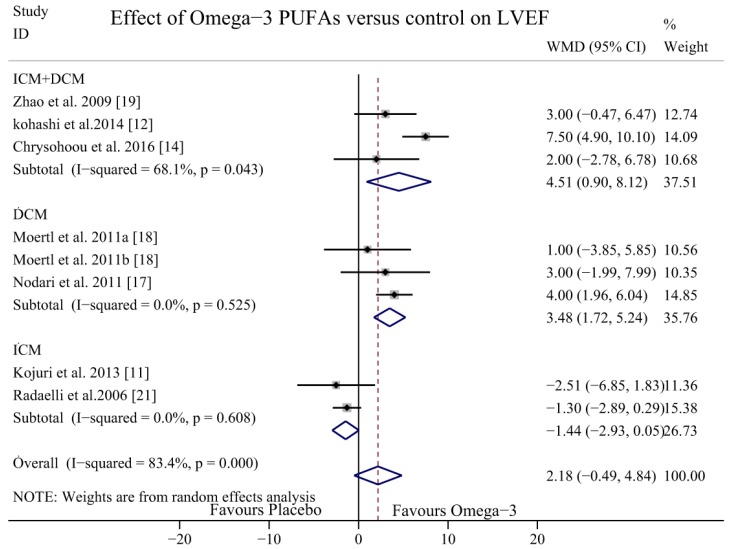
Forest plot depicting the subgroup analysis of the effect of omega-3 PUFAs on LVEF (WMD, weighted mean difference). Abbreviations: LVEF, left ventricular ejection fraction; PUFAs, polyunsaturated fatty acids.

**Figure 7:**
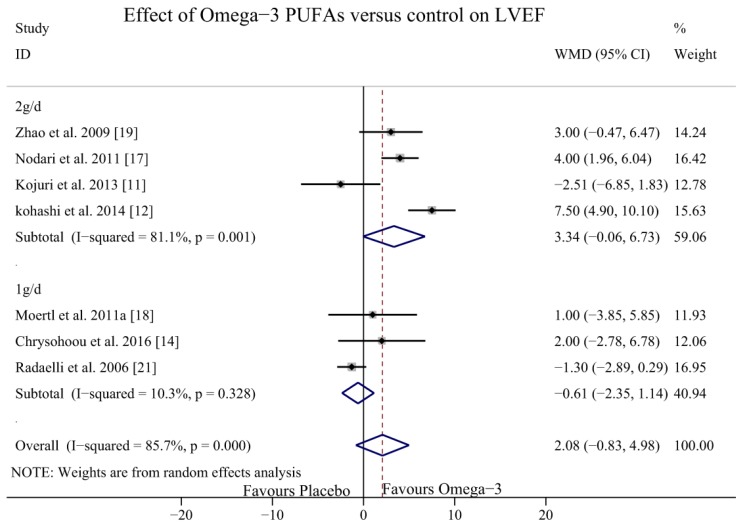
Forest plot depicting the dose-effect of omega-3 PUFAs on LVEF (WMD, weighted mean difference). Abbreviations: LVEF, left ventricular ejection fraction; PUFAs, polyunsaturated fatty acids.

**Figure 8:**
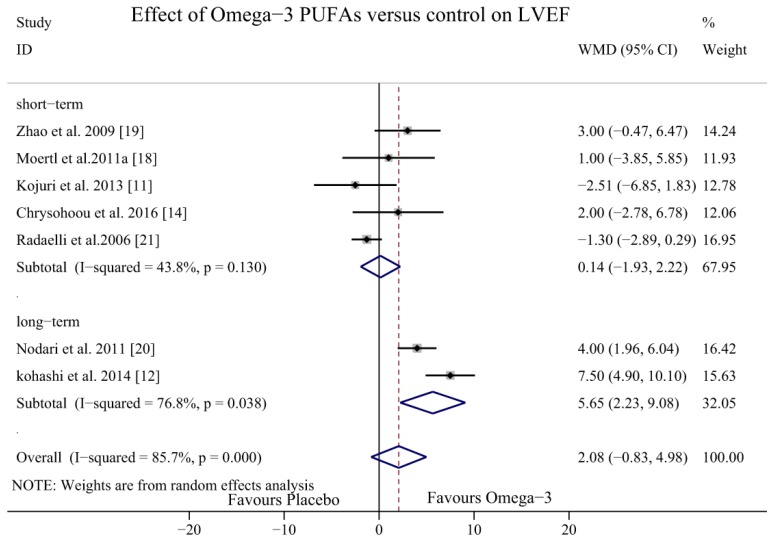
Forest plot depicting the time-effect of omega-3 PUFAs on LVEF (WMD, weighted mean difference). Abbreviations: LVEF, left ventricular ejection fraction; PUFAs, polyunsaturated fatty acids, short-term, ≤6 months; long-term, >6 months.

To explore potential sources of heterogeneity, dose-response and subgroup analysis were performed. In subgroup analyses, we classified the aetiology of HF into DCM, ICM, or both DCM and ICM together. The results of the studies that included patients with DCM and ICM showed a potential effect of omega-3 PUFAs in increasing the LVEF (WMD = 4.51, 95% CI = 0.90 to 8.12), but the heterogeneity was significant (*I*^2^ = 68.1%, *p* = 0.043). In the DCM subgroup, the LVEF was significantly increased with no heterogeneity (WMD = 3.48, 95% CI = 1.71 to 5.24; *p* = 0.525, *I*^2^ = 0.0%); however, in the ICM subgroup, the LVEF did not increase significantly with omega-3 PUFAs (WMD = −1.44, 95% CI = −2.93 to 0.05; *p* = 0.608, *I*^2^ = 0.0%) (Figure 6). In the dose-response analysis, there were no statistically significant differences between the dosage of 2 g/day (WMD = 3.34, 95% CI = −0.06 to 6.73; *p* = 0.001, *I*^2^ = 81.2%) and 1 g/day (WMD = −0.61, 95% CI = −2.35 to 1.14; *p* = 0.328, *I*^2^ = 10.3%) (Figure 7). In the sub-analysis of duration time, the follow-up group was divided into two groups: a short-term intervention group (≤6 months) and a long-term intervention group (>6 months). The LVEF was more likely to be improved in the of long-term intervention group (WMD = 5.65, 95% CI = 2.23 to 9.08, *p* = 0.038, *I*^2^ = 76.8%) but there was no significant difference in the short-term intervention group (WMD = 0.14, 95% CI = −1.93 to 2.22, *p* = 0.13, *I*^2^ = 43.8%) (Figure 8).

### 3.5. Effect of Omega-3 PUFAs on SNE Level

**Figure 9:**
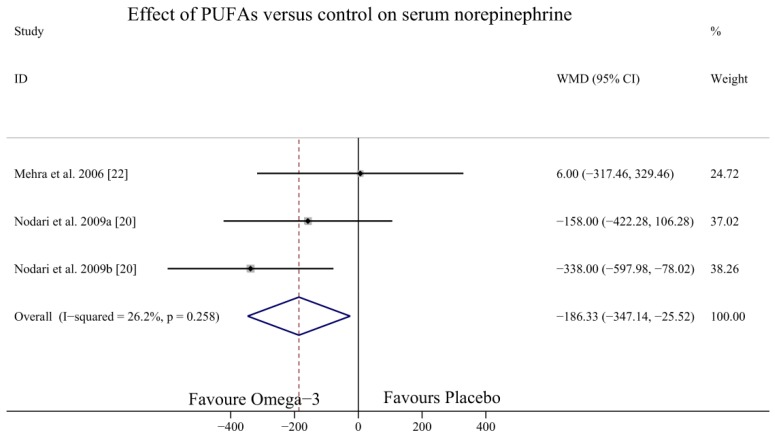
Forest plot depicting the effect of omega-3 PUFAs on SNE level (WMD, weighted mean difference). Abbreviations: PUFAs, polyunsaturated fatty acids; SNE, serum norepinephrine.

Only two studies [20,22] with three study arms investigated the effect of omega-3 PUFAs on the SNE levels of HF patients. The results indicated that omega-3 PUFAs could significantly decrease SNE levels (WMD = −186.33, 95% CI = −347.14 to −25.52; *p* = 0.258, *I*^2^ = 26.2%) (Figure 9).

### 3.6. Effect of Omega-3 PUFAs on Otherclinical Outcomes

**Figure 10:**
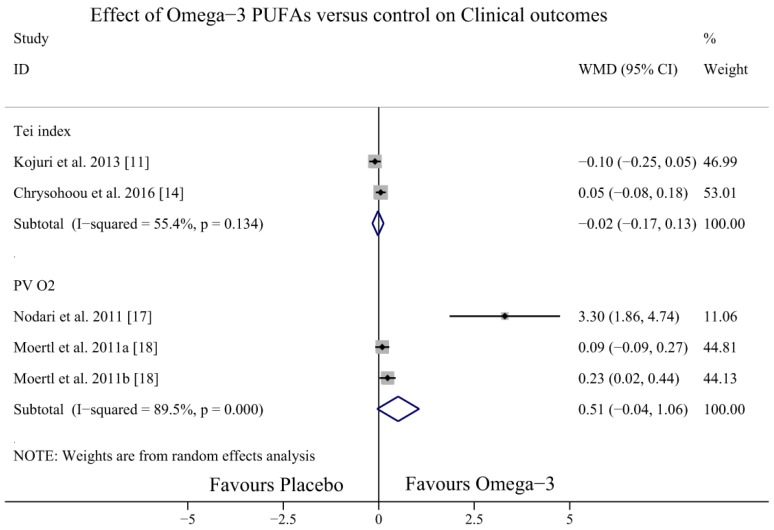
Forest plot depicting the effect of omega-3 PUFAs on Tei index and peak VO_2_ (WMD, weighted mean difference). Abbreviation: PUFAs, polyunsaturated fatty acids; peak VO_2_, peak oxygen consumption.

Four studies [11,14,17,18] investigated the effect of omega-3 PUFAs on other clinical outcomes (Tei index, peak VO_2_). The results showed no significant differences in the effect of omega-3 PUFAs on the Tei index (WMD = −0.02, 95% CI = −0.17 to 0.13; *p* = 0.134, *I*^2^ = 55.4%) and peak VO_2_ (WMD = −0.51, 95% CI = −0.04 to 1.06; *p* = 0.00, *I*^2^ = 89.5%) (Figure 10).

### 3.7. Publication Bias and Sensitivity Analysis

**Figure 11:**
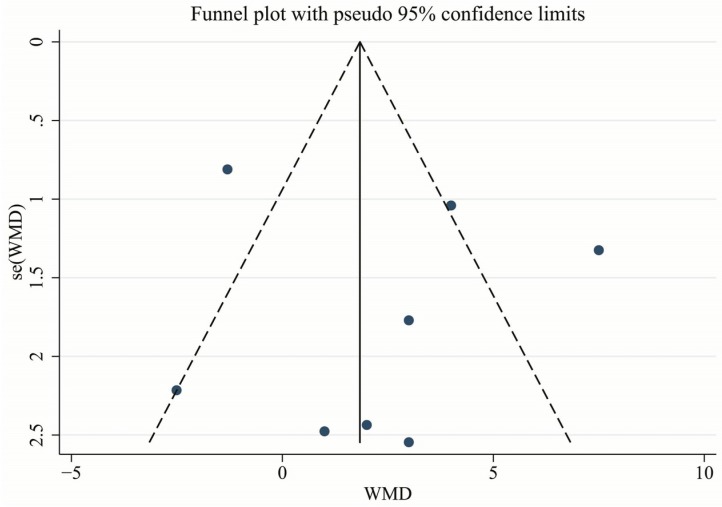
Funnel plot of the effect of omega-PUFAs on LVEF. Abbreviations: LVEF, left ventricular ejection fraction; PUFAs, polyunsaturated fatty acids.

The symmetry funnel plot of the LVEF indicated that there was no significant publication bias in the analysis (Figure 11). We also performed a sensitivity analysis by excluding individual studies one by one to evaluate the stability of our estimates. Based on the results of this analysis, there was no significant modification of our conclusions.

## 4. Discussion

Data from our meta-analysis indicated that additional omega-3 PUFA supplementation led to lower BNP and SNE levels, which would represent evidence of the benefits of omega-3 PUFAs in patients with HF. However, omega-3 PUFAs did not improve the LVEF and other clinical outcomes (Tei index, peak VO_2_) in patients with HF overall. Although the subgroup analysis showed that the LVEF increased in DCM patients and with long-time treatment, the dose-response analysis did not reach statistical significance.

The result of our meta-analysis showed that omega-3 PUFAs could decrease BNP and NT-proBNP levels. Plasma BNP or NT-proBNP concentration is an indicator of the severity of HF and increases exponentially as the cardiac condition worsens [23,24,25,26]. Linssen et al. [27] demonstrated that the doubling of NT-proBNP level was associated with a 22% increase in all-cause mortality and a 16% increase in cardiovascular events. Although the results were obtained from limited studies, based on our meta-analysis, the lower BNP level might demonstrate that omega-3 PUFAs were beneficial to HF patients. BNP levels were correlated with increased ventricular wall stress, and a beneficial reduction in wall stress can be assumed with lower BNP levels, which is in accordance with an improvement in diastolic function. In addition, adipose tissue exhibits a high density of natriuretic peptide receptors, which has an underrated role in the regulation of the fat metabolism. HF patients who are overweight or obese have a better prognosis than HF patients with normal body weight, this phenomenon is called the “obesity paradox” [28]. It was suspected that a higher PUFAs concentration could cause a better prognosis in patients with abdominal obesity [29].

The present meta-analysis showed that omega-3 PUFAs could not increase the LVEF and improve other clinical outcomes (Tei index, peak VO_2_), and this finding was not consistent with that of a previous meta-analysis [11]. However, our findings should be interpreted with caution, because the studies by Chrysohoou et al., 2016 [14] and Kohashi et al., 2014 [12] showed negative results and significant heterogeneity among the included studies. We assume that the differences in ages [30], underlying causes of HF, varying severity of HF, and higher daily doses and ratio of EPA-DHA may have caused the heterogeneity. Subgroup analyses suggested that there were increased effects for non-ischaemic HF than for ischaemic HF patients, which were in accordance with the findings of previous studies [10]. It is currently debated whether supplementation of omega-3 PUFAs in ICM patients is associated with a protective effect on major cardiovascular events. The Alpha Omega Trial, which included 4837 patients, showed that low-dose omega-3 PUFAs did not significantly reduce the rate of major cardiovascular events in post-myocardial infarction (MI) patients [31]. The study by Hoogeveen [13] also demonstrated that there were no effects of omega-3 PUFAs supplementation on NT-proBNP levels after MI. The potential mechanisms might be that excess fatty acid intake magnifies post-MI chemokine signalling and induces non-resolving overactive inflammation within cardiosplenic and cardiorenal network with aging [32]. The inflammatory response into the infarcted zone lead to migration of monocytes, stimulation of the sympathetic nervous system and renin–angiotensin–aldosterone system, and the release of natriuretic peptides [33].

However, the GISSI-Prevenzione investigators showed that the reduction of cardiac death due to supplementation with omega-3 PUFAs was most pronounced for patients with systolic left-ventricular dysfunction [9]. In the Acute Studies of Nesiritide in Decompensated Heart Failure (ASCEND-HF) trial, when the LVEF was evaluated as a continuous measure, it exhibited a U-shaped pattern with mortality [34], so modest improvement in the LVEF was barely in direct relationship with mortality. Further investigation is warranted to elucidate the effect of omega-3 PUFAs in the mortality of HF patients. Furthermore, in the analysis of the dose-effect and subgroup effects, we found that both dosages of 2 g/day and 1 g/day did not show any significant difference, but that there were significant differences in long-term treatment (12 months). More intervention studies should explore the effects of higher doses and long-term use of omega-3 PUFA supplementation in HF patients [35].

Our meta-analysis showed that omega-3 PUFAs could significantly decrease SNE levels. Previous studies indicated that the benefits of omega-3 PUFAs on cardiac function and remodelling may be mediated by multiple effects on multiple processes and mechanisms [36,37]. Omega-3 PUFAs could preserve cardiac mitochondrial function by stimulating the expression of proteins involved in cardiac lipid metabolism to reduce myocardial oxygen consumption, allowing improved mechanic efficiency of the ventricle [38], or by inhibiting inflammatory nuclear transcription factors such as TNF-α, IL-1, and IL-6 [31]. Sympathetic nervous system hyperactivity and increased synthesis and release of cardiac BNP are cardinal features of HF [39]. There is a well-recognised, direct correlation between enhanced norepinephrine levels and increased mortality in HF patients [40,41]. Some data have supported the finding of a modulating effect of omega-3 PUFAs on cardiac autonomic control, but the evidence is not conclusive [42]. Decreasing SNE levels might be one of several beneficial effects of omega-3 PUFAs in HF patients, but because of the small number of participants and short-term follow-up, large-scale clinical trials are required to confirm this benefit.

There are several limitations to the present meta-analysis that should be considered. Firstly, the small sample size and limited number of clinical trials might lead to imprecise results. Second, the changes in BNP levels, LVFF, and SNE levels are all surrogate endpoints. With few studies evaluating terminal outcomes, we were unable to perform analyses of the effects of omega-3 PUFAs on cardiovascular events and mortality in patients with HF. Thirdly, different lengths of intervention time, different doses, and different composition of omega-3 PUFAs in each study might cause a confounding bias and affect the treatment effect. Fourthly, significant heterogeneity in LVEF analyses might affect the interpretation of the true intervention effect. There were not enough studies to perform a meta-regression to explore the source of heterogeneity. Finally, in the subgroup analysis, only a few studies tested the effect of omega-3 PUFAs on patients with only ICM or DCM.

## 5. Conclusions

In conclusion, treatment with omega-3 PUFAs decreased the BNP and SNE levels and increased the LVEF in DCM patients. These findings suggest that omega-3 PUFAs might be beneficial to HF patients, but the evidence is not definitive. In the future, high-quality RCTs with large numbers of participants are needed to investigate the optimal dosage and components of omega-3 PUFAs to improve the prognostic outcomes of HF patients.

## Figures and Tables

**Table 1:** Characteristics of included randomised controlled trials (RCTs).

Author/Year	Country	Design	Sample Size Total (Control/Treatment)	Population of Heart Failure	Mean Dose of Omega 3 (g/Day)	Duration (Months)	Control	Main Indexes
Mehra et al., 2006 [22]	USA	RCT	14 (7/7)	NYHA III-IV ICM and DCM	1 (EPA/DHA)	4.5	Placebo	TNF-a, IL-1 Serum norepinphrine
Radaelli et al., 2006 [21]	Italy	RCT	25 (15/10)	post-MI EF < 40%	2 (EPA/DHA)	4	Placebo	R-R interval, NYHA class LVEF
Nodari et al., 2009 [20]	Italy	RCT	44 (22/22)	DCM EF ≤ 45%	1 (EPA/DHA)	6	Placebo	Serum norepinephrine TNF-α, IL-1, IL-6
Zhao et al., 2009 [19]	China	RCT	76 (37/38)	NYHA II-III DCM and ICM EF < 40%	2 (EPA/DHA)	3	Placebo	hs-CRP LVEF, NT-proBNP
Moertl et al., 2011 [18]	Austria	RCT	49 (16/14,13)	NYHA III-IV DCM EF < 35%	1.4 (EPA/DHA)	3	Placebo	LVEF, hsIL-6 INF-α, Peak VO_2_
Nodari et al., 2011 [17]	Italy, USA	RCT	133 (66/67)	NYHA II-III DCM EF ≤ 45%	2 (EPA/DHA)	12	Placebo	LVEF Peak VO_2_ IL-6, IL-1, TNF-α
Kojuri et al., 2013 [11]	Iran	RCT	70 (32/38)	NYHA II-III ICM EF < 40%	2 (EPA/DHA)	6	Placebo	BNP, Tei index 6-min walk test LVEF
Kohashi et al., 2014 [12]	Japan	RCT	139 (68/71)	ICM and DCM EF 37.6% ± 8%	1.8 (EPA)	12	no	LVEF NYHA class BNP hsCRP, TNF-α
Chrysohoou et al., 2016 [14]	Greece	RCT	250 (125/125)	NYHA I-III ICM and DCM	1 (EFA/DHA)	6	Placebo	LVEF BNP Tei index

Abbreviations: RCT, randomised controlled trial; HF: heart failure; NYHA, New York Heart Association; ICM, ischemic cardiomyopathy; DCM, dilated cardiomyopathy; EPA, eicosapentaenoic acid; DHA, docohexaenoic acid; ALA, plant-derived alpha-linolenic acid; TNF-α, tumor necrosis factor-α; IL-1,6, Interleukin-1,6; BNP, brain natriuretic peptide; NT-proBNP, N-terminal pro B-type natriuretic peptide; CRP, C reactive protein; MI, myocardial infarction; peak VO_2_, peak oxygen consumption.

**Table 2:** Patient characteristics.

Study	Age, Year (Treatment/Control)	Weight (kg)	Body Mass Index (kg/m^2^)	Treatment Initiation	Ratio of EPA to DHA
Mehra et al., 2006 [22]	59 ± 7/61 ± 8	58 ± 11/65 ± 11	NA	Progression of HF	44% EPA, 24% DHA and 12% other
Radaelli et al., 2006 [21]	59.4 ± 2.5/60.1 ± 2.7	NA	NA	Chronic post-MI HF	EPA/DHA ratio of 0.9 to 1.5
Nodari et al., 2009 [20]	61.09 ± 11.22/64.82 ± 9.46	NA	NA	Progression of HF	EPA/DHA of 0.9:1.5
Zhao et al., 2009 [19]	74 ± 6/71 ± 10	NA	24.7 ± 3.6/24.0 ± 2.9	Progression of HF	EPA 180 mg, DHA 120 mg
Moertl et al., 2011 [18]	58.6 ± 7.0, 61.9 ± 9.6/55.1 ± 12.7	NA	27.0 ± 3.3, 27.8 ± 5.0/28.0 ± 5.6	Progression of HF	EPA 465 mg, DHA 375 mg
Nodari et al., 2011 [17]	61 ± 11/64 ± 9	76.9 ± 10.1/76.0 ± 7.6	25.9 ± 2.3/25.7 ± 2.22	Progression of HF	EPA/DHA ratio of 0.9 to 1.5
Kojuri et al., 2013 [11]	56/58	NA	NA	Progression of HF	NA
Kohashi et al., 2014 [12]	71.4 ± 7.7	NA	22.7 ± 2.4/23.1 ± 2.9	Progression of HF	EPA
Chrysohoou et al., 2016 [14]	63 ± 12.8/63.4 ± 14.6	NA	28.72 ± 3.88/27.6 ± 4.76	Progression of HF	NA

Abbreviations: NA, not available; EPA, eicosapentaenoic acid; DHA, docohexaenoic acid; MI, myocardial infarction; HF, heart failure.

## Supplementary Materials

The following are available online at http://www.mdpi.com/2072-6643/9/1/18/s1.

## Appendix A. Search Strategy in PubMed

(“heart failure”(MeSH Terms) OR (“heart”(All Fields) AND “failure”(All Fields)) OR “heart failure”(All Fields)) AND ((“fatty acids, omega-3”(MeSH Terms) OR (“fatty”(All Fields) AND “acids”(All Fields] AND “omega-3”(All Fields)) OR “omega-3 fatty acids”(All Fields) OR “omega 3”(All Fields)) OR PUFA(All Fields) OR (“fish oils”(MeSH Terms) OR (“fish”(All Fields) AND “oils”(All Fields)) OR “fish oils”(All Fields) OR (“fish”(All Fields) AND “oil”(All Fields)) OR “fish oil”(All Fields)) OR ((“military personnel”(MeSH Terms) OR (“military”(All Fields) AND “personnel”(All Fields)) OR “military personnel”(All Fields) OR “marine”(All Fields)) AND oil(All Fields)) OR (“cod liver oil”(MeSH Terms) OR (“cod”(All Fields) AND “liver”(All Fields) AND “oil”(All Fields)) OR “cod liver oil”(All Fields)) OR (“fatty acids, omega-3”(MeSH Terms) OR (“fatty”(All Fields) AND “acids”(All Fields) AND “omega-3”(All Fields)) OR “omega-3 fatty acids”(All Fields) OR “n 3 fatty acid”(All Fields)) OR (“fatty acids, unsaturated”(MeSH Terms) OR (“fatty”(All Fields) AND “acids”(All Fields) AND “unsaturated”(All Fields)) OR “unsaturated fatty acids”(All Fields) OR (“polyunsaturated”(All Fields) AND “fatty”(All Fields) AND “acid”(All Fields)) OR “polyunsaturated fatty acid”(All Fields)) OR (“eicosapentaenoic acid”(MeSH Terms) OR (“eicosapentaenoic”(All Fields) AND “acid”(All Fields)) OR “eicosapentaenoic acid”(All Fields)) OR (“docosahexaenoic acids”(MeSH Terms) OR (“docosahexaenoic”(All Fields) AND “acids”(All Fields)) OR “docosahexaenoic acids”(All Fields) OR (“docosahexaenoic”(All Fields) AND “acid”(All Fields)) OR “docosahexaenoic acid”(All Fields)) OR EPA (All Fields) OR DHA(All Fields)).
